# Resistance Exercise Training in McArdle Disease: Myth or Reality?

**DOI:** 10.1155/2018/9658251

**Published:** 2018-09-30

**Authors:** Aleksandra Pietrusz, Renata S. Scalco, Ros Quinlivan

**Affiliations:** MRC Centre for Neuromuscular Diseases, Institute of Neurology, Queen Square, London, UK

## Abstract

McArdle disease is a metabolic myopathy mainly characterised by symptom onset during physical activities or isometric muscle contraction. Resistance (also termed strength) training is a type of physical exercise focusing on the use of resistance (e.g., lifting weights) to induce muscular contraction, which builds muscle mass and strength. Historically people with McArdle disease were advised to avoid resistance exercises and any other form of physical activity involving high mechanical loads such as prolonged isometric contraction. Paradoxically, a clinical trial exploring the benefits of strength training in this patient population was published. The theory supporting strength training relied on the use of the ATP molecule and the creatine phosphate (ATP-phosphocreatine system) as energy sources for skeletal muscles. Here, we report two patients with McArdle disease who performed weight training at local gyms. A single set of repetitions lasted for maximum 10 seconds with minimum of 30 seconds of rest period in between sets of exercises. Benefits of this type of training included improvement in quality of life and amelioration of McArdle disease symptoms. We provide further safety evidence of this type of exercise in people with McArdle disease. We emphasise the importance of using a specific protocol developed for people affected by this condition.

## 1. Introduction

McArdle disease (glycogen storage disease type V, GSDV) is a metabolic myopathy caused by homozygous or compound heterozygous mutations in* PYGM*. It is characterised by muscle glycogen phosphorylase deficiency in skeletal muscle. The lack of this enzyme impairs glycogen breakdown (glycogenolysis); thus affected people are unable to use glycogen as an energy source in skeletal muscles. People with McArdle disease experience muscle spasms and myalgia within seconds to minutes of starting physical activity. These symptoms are commonly associated with tachycardia. If the physical activity is continued when symptoms are present, it can lead to muscle contractures and muscle damage (rhabdomyolysis), which may be complicated by compartment syndrome or life-threatening emergencies such as acute renal failure. However, if the physical activity is continued in a gentle manner by slowing down or pausing and restarting to manage symptoms, after approximately 8-10 minutes the* second wind* phenomenon will occur. The* second wind* is characterised by better tolerance for aerobic exercise associated with a decrease in muscle pain and heart rate. This happens because of increased blood flow in the skeletal muscle and the ability of the body to find alternative sources of energy, like glucose released from the liver glycogen store; and fatty acids, carbohydrate, and protein metabolism via mitochondrial respiration, aerobic metabolism [1, 2].

Resistance (or “strength”) training is a form of physical exercise (e.g., lifting weights), which increases muscle mass and strength. Historically, people with McArdle disease were advised to avoid resistance exercises and any other form of physical activity involving an intense effort or high mechanical loads in a given muscle group (e.g., carrying heavy items, walking up hill) or performing isometric exercise (e.g., yoga). Those recommendations were in the interests of safety, since performing such activities in combination with an impaired glycogen metabolism would greatly increase the risk of rhabdomyolysis [3–5].

**Figure 1:**
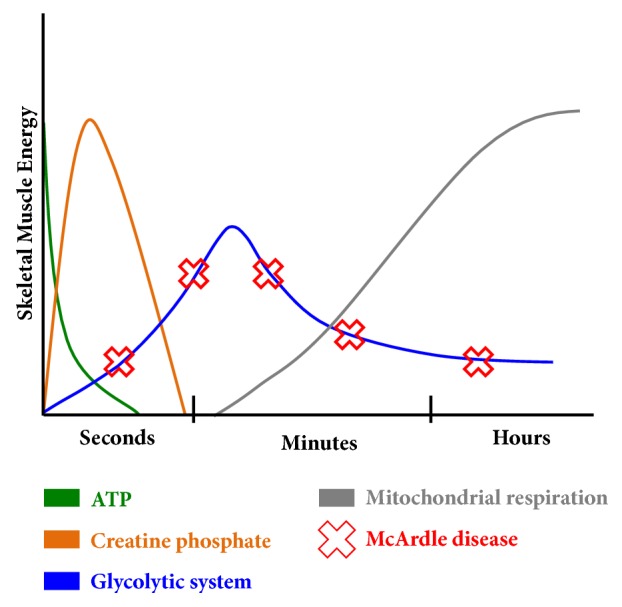
Schematic representation of the main energy sources for skeletal muscles during exercise. Red crosses represent the affected glycolytic system (glycogen) in McArdle patients.

Paradoxically, two recent publications reported people with McArdle disease performing weight lifting training safely [6, 7]. The theory supporting strength training in these patients was based upon known muscle metabolism (Figure 1). In addition to glycogen, a second short burst of energy for high intensity exercise can be obtained via the phosphagen pathway (ATP-phosphocreatine system), which allows anaerobic activities lasting up to 10 seconds at maximal exercise intensity [8, 9]. The more intense the exercise is, the faster the energy is consumed from the ATP-phosphocreatine system. It takes at least 30 seconds to partly resynthesize this energy source and around 3 minutes to fully replenish, depending on the intensity of performed exercise. As this metabolic pathway is not impaired in people affected by McArdle disease, patients may exercise safely when respecting the ~10 seconds of energy availability.

It is widely recognised that strength training in a healthy population results in significant functional benefits, including increase in bone density, improvement of cardiac function, and improvement of overall health. Other adaptations to resistance training include increased motor unit recruitment and firing rate and synchronization resulting in muscle strength improvement. Increased muscle cross sectional area (hypertrophy), hyperplasia, and increased upregulation in muscle growth hormone stimulate muscles to grow. Increased number of capillaries and mitochondria improve oxygenation and blood flow to working skeletal muscles [10]. Improved muscle strength has a carry over to everyday life activities making them easier to perform. Such benefits support further studies of weight lifting in people suffering from chronic diseases, including McArdle disease.

Here we present two people with McArdle disease who have been performing resistance training without experiencing McArdle-related injuries. Both showed positive results, such as increased strength, muscle hypertrophy, fat loss, and attenuation of McArdle symptoms, which substantially diminished after starting this form of training.

## 2. Case Series

### 2.1. Patient 1

A 37-year-old man reported exercise/activity-related muscle pain and fatigue from early childhood. His symptoms were labelled as “growing pains” by different medical professionals, and he was often called “a lazy child”. He had difficulties to keep up with his friends and family when walking. He reported physical education classes and school games as bad experiences. Throughout his life he continued to avoid activities that provoked muscle symptoms. Despite not being aware of the* second wind* phenomenon, he used strategies such as slowing down or stopping and restarting when symptoms eased off. He reported pain in his muscles within a few minutes or sometimes seconds of initiating physical activity, particularly noticeable when walking upstairs, walking up hills, and carrying shopping bags. He had a previous medical history of four episodes of myoglobinuria triggered by playing football or lifting heavy items. He was diagnosed with McArdle disease at the age of 20 years based on an abnormal muscle biopsy. He was later confirmed to have a homozygous mutation (p.Gln567Pro) in* PYGM*.

Physical examination at the age of 29 revealed rounded shoulders with hypertrophy of deltoid, biceps, and calf muscles. He had significantly wasted pectoralis muscles and bilateral scapular winging, but muscle strength was normal. When diagnosed he had been advised to complete at least three sessions of walking 30 minutes per week. However, he did not change his physical activity levels and did not report changes in his quality of life.

After graduation he started his first office job. He became more sedentary, his weight increased, and symptoms worsened. He reported difficulties in walking short distances. Everyday tasks such as vacuuming and cutting grass became more difficult.

He joined a local gym, where he has been a member for approximately 9 years. Initially exercises included walking on a treadmill and cycling on a stationary bike. He tried resistance machines but was not confident in using them. Four years ago, he approached a personal trainer, who took the time to learn about his metabolic condition [11]. He suggested that weight lifting could be safe and effective if using principles of strength training after considering the pathophysiology of McArdle disease.

Initial phase of training consisted of gentle 15-20 minutes aerobic exercise to warm up and get into* second wind *(walking on a treadmill, cycling on a stationary bike) followed by learning strengthening exercise techniques using body weight and very light weights. Training intensity gradually progressed towards mobility movements (e.g., Turkish get ups, walking lunges), increasing resistance as well as adding high intensity interval training (HIIT) protocol on the rowing ergometer at the end of the session. Strength exercises were mainly performed using compound movements with free weights rather than resistance machines. Currently, he performs a 15-20 minute aerobic warm up. He performs 1-5 repetitions with 2-5 minutes rest in between sets depending on the % of one repetition max (1RM). He also tried a different protocol involving four repetitions with 30 seconds rest followed by another four repetitions of the same weight. He has been doing two sessions with the personal trainer and two sessions on his own each week. When without the trainer, he only performs exercises he is familiar with.

Over the past four years of strength training his weight increased from 65kg to 70kg; body composition dramatically changed by significantly increasing muscle bulk, in particular of his quadriceps, gluteus, pectoralis, deltoids, and trapezius muscles. His waist remained the same; collar size increased from size 14.5 to 15.5/16.0. He had to purchase new clothing due to dramatic change in body composition. Importantly, his muscle strength increased significantly as described in Table 1.

He also performed other exercises, including lateral pull downs, TRX rows, TRX pull-ups, body weight pull-ups from jumps, Olympic lifting movements, box jumps, medium height approx. 45cm, and pistol squats.

He has never experienced any McArdle symptoms during or after strength training and has not had myoglobinuria following his gym sessions. His serum CK level varied as expected in McArdle disease, with a decreasing trend (average CK in 2011-2014: 3,006 IU/L, average in 2015-2017: 1,029 IU/L; last measured in July 2017: 941 IU/L; reference range: up to 240 IU/L). Improvement in McArdle symptoms was described as a delayed onset of skeletal muscle symptoms, which now occurs at much higher physical activity intensity. Reaching the* second wind* is more efficient. In general, his quality of life improved significantly. He has been eating high protein diet with a bigger portion of carbohydrates on training days. He autonomously chose not to take any supplements containing glucose pre- or intra-training sessions.

### 2.2. Patient 2

A 46-year-old man also reported exercise/activity-related pain and fatigue from early childhood. As patient 1, he was always considered to be “a lazy child”. He was not able to run and physical activities such as walking or swimming were challenging. As a child he reported trying to build a good relationship with his physical education teachers, so they would feel pity for him and he could avoid any strenuous physical activities (PA). He experienced three severe episodes of rhabdomyolyses in his life. The first one happened during childhood, which followed vigorous physical activities. The second rhabdomyolysis episode was at the age of 18 following multiple squats, which he performed during a physical test for military service. Not qualifying for the military service, instead he had to complete a civilian service at the university hospital at the age of 22. As he was clearly weaker than his colleagues, a diagnostic investigation took place, which included a skeletal muscle biopsy. At that time, doctors advised him to avoid excessive physical effort. He was told about the* second wind* phenomenon; however, it was not explained how to reach it. Additionally, he was recommended to eat a maximum of 20% of his daily food intake in carbohydrates. However, the rest of the macronutrients recommendations were not specified. In the following years, his physical activity level decreased, leading to physical deconditioning, loss of muscle mass, and increase in fatty adipose tissue and body weight.

The third rhabdomyolysis episode was experienced one year ago and was nearly fatal. He was undergoing physical assessment for the insurance company and pushed himself too hard, resulting in a severe contracture of his lower back muscles. He was admitted to hospital. During the hospital stay he was also diagnosed with myocarditis. Following this episode, he decided to learn more about the condition to manage it better.

He began doing gentle aerobic exercises, which improved his ability to attain a* second wind*. He attended a conference where he met a Spanish team from whom he learnt about the strength training trial performed in Madrid and decided to try it himself in a local gym [7]. Initially he approached a personal trainer. However, he did not feel the trainer understood his condition and was pushing him too hard. He decided to write his own programme based on strength training principles and exercises he learnt at the conferences and meetings.

**Figure 2:**
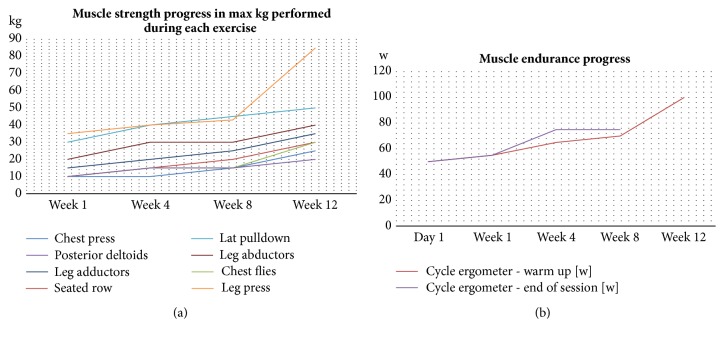
Training progress in patient 2. (a) Muscle strength progress; (b) muscle endurance progress using a stationary bicycle (measured in watts).

When in the gym he reports always doing an aerobic “warm up” by cycling on a stationary bike for 20 minutes, he then exercises on resistance machines (chest press, seated row, butterfly, chest horizontal adduction, reversed butterfly, rare deltoids and trapezius, lat pulldown, leg press, leg adductors, and leg abductors). He finishes his sessions with more aerobic exercise by walking on a treadmill and/or cycling on a stationary bike.  Figure 2 and Table 2 illustrate his progress during the first three months of training (based on personal records). He completes between 5 and 15 repetitions of each exercise with one-minute rest in between sets. He stops a set of exercise earlier if he feels any discomfort in the muscle.

He did not report Delayed Onset Muscle Soreness (DOMS) or McArdle symptoms following initial sessions. After three months of resistance training, he found his sleep pattern, overall stamina and McArdle symptoms improved. He is now able to walk two kilometres stopping only once, as opposed to previously when he would have to stop multiple times. He has also found it easier to perform everyday tasks such as changing a car tyre. He has been eating a balanced diet. He autonomously chose not to take any supplements containing glucose pre- or intratraining session.

## 3. Discussion

Here we report two people with McArdle disease who have safely performed resistance training. The benefits were not only restricted to an improvement in muscle strength; it also appears to have helped their underlying metabolic myopathy, as both patients reported a delayed onset of McArdle symptoms occurring at a much higher intensity of physical activity.

Owing to the risk of recurrent rhabdomyolysis, for many years resistance training was strongly warned against in people with McArdle disease. The first case report suggesting a safe protocol of resistance training in McArdle was published in 2013 in Spain [6]. A 14-year-old male with McArdle disease performed supervised, light to moderate-intensity resistance training program for a period of six weeks (two sessions weekly). Training resulted in a significant increase in muscle strength with no severe adverse events. The reported patient was reclassified to a lower disease severity class as he became asymptomatic considering exercise limitations. The authors' preliminary data suggested that supervised, light to moderate resistance training was feasible in a teenager with McArdle disease with potential clinical benefits. Following this publication, a clinical trial was conducted in Spain by Santalla et al. (2014) showing beneficial effects of a four-month resistance training program in seven adults with McArdle disease (five female) [7]. The reported benefits included increased lean muscle mass (assessed by DEXA scan) and a gain in muscle strength, both of which then significantly deteriorated during a 2-month detraining period. All the participants reported improvement in their McArdle symptoms. Compliance with the training protocol was ≥84% in all patients and no major complications occurred. In both Spanish studies, glucose intake before resistance exercise was part of the training protocol.

Our case series has confirmed the findings reported by the Spanish researchers. This short report of two patients provides additional evidence for the safety and benefit of strength training in McArdle disease when a correct training protocol is followed. Benefits include increased muscle strength and muscle mass and improvement in severity of McArdle symptoms and quality of life. No McArdle-related adverse events or exercise related injuries and importantly no signs of acute rhabdomyolysis were reported by either of the patients.

Patient 1 has been following strength training for four consecutive years. He initially followed the guidance of a local personal trainer, who learnt about McArdle disease and understood the underlying disease pathophysiology. Patient 1 did not have any knowledge of resistance training prior to his personal trainer's recommendations. Patient 2 took the initiative to learn more about McArdle disease following a life-threatening episode of rhabdomyolysis; he attended multiple workshops, conferences, and patients' meetings, where he first learnt about resistance training. For safety reasons, both patients performed the resistance training after reaching the* second wind*. This agrees with the previously published clinical trial [7]. However, our patients did not take glucose drinks prior to and during training sessions, and they followed a healthy, balanced diet. Patient 1 increased carbohydrate intake during training days to improve his energy levels before and during training session, as well as recovery after training.

Considering that the ATP-phosphocreatine energy system pathway is not affected in people with McArdle disease, we have demonstrated in our small case series that this population can safely exercise at a high intensity level for up to 10 seconds at time, depending on training intensity, with 30 seconds to 3 minutes break in between sets of exercise in order to allow energy to be restored. However, strength training in people with McArdle disease should only be performed following an aerobic warm up to put the patient into* second wind* to enable working muscle to have access to all available energy sources and for better recovery in between short bouts of resistance exercises. An important point is that, for safety, we recommend that anyone with McArdle disease interested in performing resistance training should speak with their specialist medical team before starting such a program of training.

In healthy people, DOMS may be experienced after initial sessions and when their training mode is changed. We would also expect this to occur in people with McArdle disease. Further studies should be performed to understand and describe the differences between DOMS symptoms and McArdle-related myalgia, as this knowledge may help people living with the condition.

Strength training results in a decrease in muscle damage with repeated resistance exercise exposure [10]. Therefore, considering the disease mechanisms of McArdle disease, this type of training may have potential to increase resistance of the muscle fibres to damage during physical activity, decreasing the risk of recurrent myoglobinuria and rhabdomyolysis. Also, with increased muscle strength, people with McArdle disease are likely to be able to function to a higher physical activity level before experiencing symptoms.

Interestingly, poor bone mass (BM) starting in early adulthood was recently shown to be associated with McArdle disease [12]. It was reported that both active and nonactive people with McArdle disease showed compromised bone health. However, patients participating in more physical activities seem to have a healthier body composition phenotype (higher lean muscle mass and bone mass), with active patients showing higher lean muscle mass compared to their sedentary patient peers. It is known that participating in regular physical activity improves bone strength [13, 14]. Also, land-based weight-bearing activities increase BM more than nonweight-bearing activities (e.g., swimming) in weight-loaded skeletal regions [15, 16]. This new published scientific data provides an additional rationale to support implementation of strength training intervention in these patients.

## 4. Conclusions

Our report indicates that strength training appears to be safe with associated health benefits when performed appropriately for this patient population; i.e., short bursts of resistance activity lasting no longer than 10 seconds preceded and followed by 30 seconds to 3 minutes rest. Following their exercise programs, the two patients reported improved quality of life. Educating people with McArdle disease about strength training principles, the rationale behind them, and the appropriate exercise techniques is crucial before commencing this type of training.

Currently there is no pharmacological treatment for McArdle disease. Strength training can be an effective treatment for McArdle disease when performed carefully. It changes body composition positively, increases muscle strength, and greatly benefits patients' activities of daily living and patients' quality of life.

## Figures and Tables

**Table 1:** Progress in strength of patient 1 in four years of training. All exercises consisted of 1-5 repetitions. First week: initial training program; four years of training: current training program.

**Type of Exercise**	**First week**	**Four years of training**
Deadlift	20kg	140kg

Squat	Body weight	120kg

Bench press (barbell)	15kg	50-60kg

Shoulder press (barbell)	10kg	40kg

Rows (laying prone on the bench – chest supported)	Barbell – 10kg	Barbell – 40kg
	Dumbbell – 6kg	Dumbbell – 28kg

**Table 2:** Increase in training workload in patient 2.

**Exercise**	**Day 1**	**Week 1**	**Week 4**	**Week 8**	**Week 12**
Treadmill walking (speed in km/h)	3.5	3.5	4.0	4.5	4.7

Treadmill walking (incline in %)	1.5	1.5	1.5	1.5	3.5

## Abbreviations

DOMS: Delayed Onset Muscle Soreness
GSDV: Glycogen storage disease type V
HIIT: High intensity interval training.

## Additional Points

*Learning Points. *(i) Strength training can be performed by people with McArdle disease only if specific protocols are followed. (ii) Strength training, when performed respecting McArdle disease limitations, may result in functional and quality of life benefits. (iii) It is important to warm up and to reach* second wind* before performing any type of resistance exercise in McArdle disease. (iv) Each muscle group included in the resistance exercise workout must be in* second wind.* (v) There is approximately a 10 seconds window to safely perform a single set of repetitions preceded and followed by a rest period of between 30 seconds and 3 minutes.
